# Supplementary material for: Affiliation in times of pandemics: Determinants and consequences
Source: PLoS One. 2024 Oct 31;19(10):e0306310. doi: 10.1371/journal.pone.0306310 (PMC11527318; doi:10.1371/journal.pone.0306310)
Supplement: S1 Text — (PDF) [file pone.0306310.s001.pdf]

S1 Text. Detailed Statistical Procedure

Analysis Plan

We used Structural Equation Modeling (SEM) to determine the structure of relations between all study variables listed in Table 1 (main text). The first step was to conduct a confirmatory factor analysis to evaluate the only two-factor structure measurement model, adapted from the SCO scale (Gibbons & Buunk, 1999). The second step was to conduct a structural model including all study variables. Values of CFI > .90 and RMSEA from .05 to .08 indicated acceptable model fit, RMSEA < .05 and indicated good fit, and RMSEA from .081 to .100 indicated marginal fit (Kenny, 2020; MacCallum et al., 1996). Reliability was assessed using the Composite Reliability Ω index, similar to Cronbach's alpha, and well suited for SEM (Hayes & Coutts, 2020). As a third step, a series of measurement invariance analyses were conducted to compare the gender-related variations for the standardized latent means of all constructs and their relations.

Measurement and Full Structural Models

A confirmatory factor analysis was conducted to determine the two-factor structure of SCO (Gibbons & Buunk, 1999). The CFA procedure was based on maximum likelihood estimation and was conducted using the Lavaan package (Rosseel, 2012) version 0.6-3 in R software (R Core Team, 2019) version R-4.0.2.

The remaining constructs (latent variables) were Perceived Vulnerability (items a and b), Perceived Illness (items e and f), Threat-Related Emotions (items c and d), Affiliation (items s, t and u), Intentions and Behavior regarding the lockdown (items j, k, l and g, h, i, respectively) and the use of protective measures (items m n, o, and p, q, r, respectively) were inspired from Ajzen (2002). A maximum of three items per construct was used to maintain the questionnaire

at a reasonable length. Most models were just-identified (Bollen & Hoyle, 2012) as a result of this configuration, precluding their separate CFA analyses. Instead, the factor loadings were obtained from the full structural model and consistency for each construct was examined using the composite reliability Ω index.

Power calculation

Following the practical recommendation on sample size for structural equation modeling by Kenny (2020), a 5 to 1 ratio of sample size to the number of free parameters is suggested (Bentler & Chou, 1987). The total number of elements in the initial covariance matrix is $k(k+1)/2$, where k equals the number of observed variables in the matrix. Here, the total number of parameters is (32*33)/2 = 528. The parameters that have to be estimated in the model are 32 observed variable variances, 32 loadings, 11 latent variable variances, 14 regression paths, and 5 latent variable covariance, which results in 94 free parameters and 528-94 = 434 degrees of freedom. For single group analysis, the degrees of freedom are 434 and the minimum required sample size equals to 5 (ratio)*94 (number of free parameters)*1 (number of groups) = 470. As we included a 2-group measurement invariance analysis, the minimum sample size required for the measurement invariance analysis is N = 940.

Measurement Invariance Procedure

Measurement invariance allows determining the extent to which each construct is similarly assessed in two groups of individuals by comparing levels of measurement invariance (Putnick & Bornstein, 2016; Vandenberg & Lance, 2000). More precisely, it ensures that any group difference on latent means (constructs) or regression/correlation coefficients is not due to a different assessment or distinct conceptual interpretation of the constructs by achieving a ‘scalar invariance’ where items’ factor loadings and intercepts are constrained to be equal

across groups. This allows to examine unbiased construct differences across groups. The procedure compares configural invariance (i.e., an unconstrained model) to metric (weak) invariance (i.e., factor loadings are constrained to be equal), to scalar (strong) invariance (i.e., factor loadings, intercepts are constrained to be equal). An additional step can be conducted, residual invariance (i.e., also known as strict invariance where factor loadings, intercepts and residuals are constrained to be equal across groups, see Putnick & Bornstein, 2016). However, residual invariance is not a prerequisite for testing mean group differences (Vandenberg & Lance, 2000) and therefore, was not performed in the present study.

Models' Statistics

We here present the statistics (fit indices, factor loadings and composite reliability Ω) for the SCO measurement models and the full structural models for Sample 1, 2, 2M and 2W. Results concerning the structural relations themselves are described in the main text (Results section).

Samples 1W

Social Comparison Measurement Model. The measurement model for SCO was conducted in Sample 1W. The CFA indicated an inadequate fit with all items of the SCO scale ($\chi2$ = 466.509, df = 43, CFI = .903, RMSEA = .109). Factor loadings values were the lowest for item 7 (.41) and reversed item 11 (.40) which were therefore removed (Table 1, main text). Note that the reversed item 5 (.65) loaded very poorly in Sample 2 (see below), therefore, to maximize comparability across samples, the three items were excluded. We re-runned the CFA without the three items, which improved the fit ($\chi2$ = 116.932, df = 19, CFI = .971, RMSEA = .079). Factor loadings ranged from .69 to .86 for the ability dimension, and from .68 to .88 for the opinion dimension. The two dimensions were highly correlated (r = .65, p < .001).

Composite reliability was respectively Ω = .88 and Ω = .88 for the ability and opinion dimensions.

Full Structural Models. The full mediation structural model for Sample 1W included the two highly correlated dimensions of the SCO under a second order SCO latent variable (i.e., global SCO). The mediation structural model showed good fit, $\chi2$ = 944.045, df = 434, CFI = .958, RMSEA = .040. However, one factor loading exceeded 1 due to negative variances on two items, 'fear' (c) and the 'perceived presence of symptoms' (f). Item 'anxiety' (d), rather than 'fear' (c), was removed for lower factor loading (.94 > .44). These issues were not found for Samples 2W and 2M (see below). After removing the items (d) and (f) and rerunning the Sample 1W model with Perceived Illness and Threat-Related Emotions estimated using one item each, $\chi2$ = 809.061, df = 335, CFI = .960, RMSEA = .041), the standardized factor loadings were all significant and below 1. Importantly, the structure of relations in Sample 1W between Threat-Related Emotions, Perceived Illness and Affiliation were largely replicated in Sample 2W using the removed items (cf. Results section, main text), ruling out possible differences in the hypothesized relations due to the missing items. The factor loadings ranged from .72 to .51 for Perceived Vulnerability (Ω = .55), 86 and .75 for global SCO (Ω = .79), .52 to .71 for Affiliation (Ω = .65), .91 to .95, for Lockdown Intentions (Ω = .95), -.42 to .86 for Lockdown Behaviors (Ω = .62), .68 to .99 for Protective Measures Intentions (Ω = .91), and from .62 to .90 for Protective Measures Behaviors (Ω = .84).

Samples 2, 2M and 2W

Social Comparison Measurement Model. The measurement model for SCO was conducted in the whole Sample 2. The CFA indicated an inadequate fit for the SCO scale ($\chi2$ = 489.550, df = 43, CFI = .928, RMSEA = .099). Factor loadings values were unacceptable for the two reversed items 5 (.25) and 11 (.09). As item 7 loaded poorly in Sample 1W (see above), for greater comparability with the analyses conducted in Sample 1W, we removed these three

items and re-ran the CFA, which improved the fit ($\chi2$ = 150.212, df = 19, CFI = .976, RMSEA = .081, although the RMSEA value remained relatively high, calling for cautious interpretations of the structural model. Factor loadings ranged from .75 to .84 for the ability dimension, and from .83 to .89 for the opinion dimension. Composite reliability was Ω = .90, and Ω = .89, respectively. The two dimensions were highly correlated (r = .75, p < .001).

Full Structural Models. The full mediation structural model in Sample 2 included the two highly correlated dimensions of the SCO under a second order SCO latent variable (i.e., global SCO). The model showed good fit, $\chi2$ = 1290.809, df = 416, CFI = .956, RMSEA = .045. Standardized factor loadings were all significant and globally more consistent than those observed for Sample 1W. They ranged from .68 to .78 for Perceived Vulnerability (Ω = .69), .76 to .77 for Perceived Illness (Ω = .74), .64 to .89 for Threat-Related Emotions (Ω = .75), 87 and .87 for global SCO (Ω = .86), .69 to .88 for Affiliation (Ω = .85), .93 to .95 for Lockdown Intentions (Ω = .95), -.53 to .82 for Lockdown Behavior (Ω = .68), .86 to .97 for Protective Measures Intentions (Ω = .95), and from .67 to .84 for Protective Measures Behavior (Ω = .81).

The full structural model for men in Sample 2M showed good fit, $\chi2$ = 910.070, df = 416, CFI = .953, RMSEA = .048, and comparable factor loadings (cf. Table 1, main text) and composite reliability scores (not reported). The full structural model for women in Sample 2W also showed good fit, $\chi2$ = 891.279, df = 416, CFI = .951, RMSEA = .047, with comparable factor loadings (cf. Table 1, main text) and composite reliability scores (not reported).

Measurement Invariance Statistics across Samples 2M and 2W

A multigroup measurement invariance analysis was conducted to examine gender-related variations for the constructs under interest in the present study. Measurement invariance tests indicated that the model was strongly invariant across gender groups as shown by ΔCFI and ΔRMSEA values below standard thresholds (ΔCFI = .01 and ΔRMSEA = .015, Chen, 2007; Cheung & Rensvold, 2002). As shown in Table S1, deltas were below thresholds when

comparing the configural and the metric invariance models, and then, when comparing the metric and the scalar invariance models. Achievement of scalar invariance enabled latent mean comparisons (Putnick & Bornstein, 2016; Vandenberg & Lance, 2000). Latent means and regression coefficients group comparisons (cf. Result section, main text) were conducted under scalar invariance. A false discovery rate (FDR) correction for multiple comparisons was applied to each between-sample structural comparison using the Benjamini-Hochberg method (Benjamini & Hochberg, 1995). The FDR correction rejects the *p* values exceeding a critical 5% false discovery rate threshold associated with each *p* value, considering the number of comparisons and the proportion of significant tests.
