# Supplementary material for: Affiliation in times of pandemics: Determinants and consequences
Source: PLoS One. 2024 Oct 31;19(10):e0306310. doi: 10.1371/journal.pone.0306310 (PMC11527318; doi:10.1371/journal.pone.0306310)
Supplement: S1 Table — (PDF) [file pone.0306310.s002.pdf]

S1 Table. Descriptive Statistics for All study Variables in Samples 1W, 2M and 2W

		Sample 1W (N = 827)		Sample 2M (N = 512)		Sample 2W (N = 526)		ANOVA	
	Variables	*M*	*SD*	*M*	*SD*	*M*	*SD*	*F*-Value Gender (2M vs 2W)	p^1
1	SCO ability item1	3.190	1.360	3.170	1.301	3.120	1.343	.346	.594
2	SCO ability item 2	3.260	1.410	3.300	1.291	3.210	1.390	1.261	.325
3	SCO ability item 3	3.360	1.381	3.160	1.295	3.030	1.369	2.241	.209
4	SCO ability item 4	3.410	1.492	3.090	1.345	2.960	1.393	2.062	.217
6	SCO ability item 6	3.260	1.415	3.150	1.317	2.940	1.355	6.595	**= .021**
8	SCO opinion item 8	4.270	1.171	3.650	1.231	3.600	1.333	.484	.539
9	SCO opinion item 9	3.680	1.328	3.440	1.251	3.340	1.329	1.415	.307
10	SCO opinion item 10	3.740	1.263	3.410	1.269	3.330	1.323	.878	.401
a	Perceived risk at home	3.240	1.533	3.170	1.387	3.210	1.319	.196	.680
b	Perceived risk for oneself	2.810	1.314	2.800	1.743	2.980	1.682	2.633	.171
c	Fear level during lockdown	3.400	1.731	3.700	1.737	4.140	1.790	15.831	**< .001**
d	Anxiety level during lockdown	3.700	1.862	3.760	1.708	4.080	1.763	8.604	**= .007**
c’	Fear level before lockdown	2.420	1.514	2.560	1.494	2.660	1.499	2.872	.155
d’	Anxiety level before lockdown	3.360	1.829	3.140	1.608	3.310	1.575	1.094	.353
e	Probability of you being infected	2.830	1.290	2.600	1.351	2.480	1.198	2.030	.217
f	Probability of presenting symptoms	.520	.922	.660	1.149	.540	1.061	3.376	.120
g	Degree of compliance with the lockdown	5.680	.702	5.160	1.098	5.440	.915	18.743	**< .001**
h	Number of unauthorized excursions	.070	.416	.360	.953	.140	.646	18.176	**< .001**
i	Percent confined time	4.310	.665	3.970	.980	4.080	.921	3.795	.101
j	Intentions to limit excursions	5.420	.900	5.020	1.181	5.250	1.062	1.492	**= .003**
k	Intentions to comply with the lockdown	5.550	.800	5.070	1.168	5.350	1.007	17.543	**< .001**
l	“I will respect the lockdown”	5.510	.821	5.080	1.116	5.320	.982	13.686	**< .001**
m	“As long as the authorities .. so”	5.550	.775	5.110	1.139	5.420	.899	24.165	**< .001**
n	“I intend to comply with the use of protective measures”	5.580	.736	5.110	1.149	5.450	.869	29.544	**< .001**
o	“I will respect the use of protective measures”	5.350	1.056	5.040	1.146	5.340	1.012	19.675	**< .001**
p	Average time spent using protective measures	3.880	1.410	3.730	1.508	4.050	1.425	11.833	**.003**
q	“Since the lockdown, I have always used the protective measures”	5.070	.981	5.000	1.092	5.200	.960	9.992	**.005**
r	Percent of compliance with protective measures	4.300	.832	4.040	1.056	4.310	.923	19.938	**< .001**
s	Time spent on social media	1.470	1.290	1.320	1.477	1.330	1.443	.001	.976
t	Time spent on the phone	1.130	.891	1.250	1.242	1.170	1.065	1.392	.307
u	Time spent on internet communication media (Skype etc.)	.640	.836	.840	1.331	.620	1.051	8.918	**= .007**

Note. p^1 : all significant p values survived the FDR correction
