# Supplementary material for: Affiliation in times of pandemics: Determinants and consequences
Source: PLoS One. 2024 Oct 31;19(10):e0306310. doi: 10.1371/journal.pone.0306310 (PMC11527318; doi:10.1371/journal.pone.0306310)
Supplement: S2 Table — (PDF) [file pone.0306310.s003.pdf]

S2 Table. Pearson Correlation Matrix for All Study Variables in Sample 1W

	1	2	3	4	6	8	9	10	a	b	c	d	c'	d'	e	f	g	h	i	j	k	l	m	n	o	p	q	r	s	t	u
1	-																														
2	.634***	-																													
3	.528***	.723***	-																												
4	.503***	.660***	.638***	-																											
6	.515***	.590***	.587***	.628***	-																										
8	.352***	.334***	.358***	.302***	.349***	-																									
9	.424***	.463***	.504***	.402***	.451***	.680***	-																								
10	.354***	.427***	.458***	.380***	.378***	.516***	.574***	-																							
a	.041	-.019	-.022	.002	.028	.022	-.001	.014	-																						
b	-.111**	-.161***	-.163***	-.133***	-.120**	-.071	-.056	-.066	.368***	-																					
c	.161***	.189***	.174***	.200***	.180***	.113**	.151***	.137***	.210***	.185***	-																				
d	.224***	.267***	.258***	.294***	.245***	.117**	.184***	.163***	.138***	.033	.630***	-																			
c'	.100**	.132***	.135***	.181***	.134***	.079*	.058	.092**	.120**	.187***	.522***	.371***	-																		
d'	.169***	.268***	.249***	.308***	.235***	.125***	.133***	.137***	.073	.038	.374***	.653***	.567***	-																	
e	.065	.024	.025	.002	.008	.023	-.027	-.022	.043	.037	.126***	.065	.130***	.103***	-																
f	.043	.015	-.005	.011	.023	.020	-.039	-.003	.002	.032	.108**	.079	.107**	.131***	.625***	-															
g	-.063	-.002	-.018	.021	-.019	-.007	-.029	-.010	.048	.082*	.073	.017	-.015	-.031	-.050	-.019	-														
h	.063	.035	-.001	.039	.017	.142***	.066	.080*	-.037	-.078	-.020	.056	.010	.062	.043	.042	-.363***	-													
i	.012	.052	.063	.094**	.056	-.024	-.011	-.016	.049	.047	.082*	.061	.061	.056	-.023	-.004	.422***	-.121***	-												
j	-.082*	-.069	-.018	-.034	-.058	-.092**	-.020	-.025	.059	.099**	.040	-.043	-.039	-.052	-.088*	-.062	.652***	-.387***	.365***	-											
k	-.010	-.003	.031	.039	.020	.005	.046	.018	.066	.052	.063	-.021	.012	-.002	-.065	-.040	.665***	-.336***	.391***	.838***	-										
l	-.021	-.019	.019	.019	-.011	-.031	.030	.007	.051	.042	.058	-.022	-.023	-.037	-.090**	-.044	.665***	-.340***	.375***	.866***	.884***	-									
m	-.086*	-.068	-.077	-.114**	-.048	.003	-.015	-.060	.014	.047	.016	-.043	-.075	-.071	-.025	-.046	.334***	-.141***	.174***	.409***	.370***	.406***	-								
n	-.077	-.057	-.080*	-.105**	-.059	.003	-.012	-.062	.005	.044	.007	-.039	-.074	-.069	-.031	-.044	.340***	-.131***	.172***	.392***	.361***	.393***	.951***	-							
o	-.050	-.028	-.035	-.038	-.003	.011	.005	.022	.000	.017	.008	-.031	-.083*	-.055	-.080*	-.104**	.263***	-.118**	.115**	.304***	.274***	.328***	.670***	.641***	-						
p	-.144***	-.117***	-.124***	-.131***	-.124***	-.058	-.023	-.085*	.062	.153****	.042	.019	-.019	-.038	-.007	.001	.251***	-.167***	.105**	.260***	.210***	.205***	.363***	.354***	.234***	-					
q	-.158***	-.152***	-.186***	-.172***	-.151***	-.043	-.053	-.096**	.009	.105**	-.021	-.048	-.043	-.074	-.072	-.022	.319***	-.074	.135***	.324***	.287***	.281***	.575***	.558***	.392***	.543***	-				
r	-.139***	-.111**	-.185***	-.151***	-.157***	-.056	-.078*	-.093**	.009	.097**	-.032	-.056	-.070	-.086*	-.021	-.007	.310***	-.067	.232***	.313***	.272***	.265***	.507***	.487***	.328***	.556***	.766***	-			
s	.083*	.092**	.091**	.073	.097**	.049	.059	.055	.085*	.032	.124***	.140***	.040	.057	.051	.011	.010	.037	.045	.040	.034	.034	.068	.055	.001	-.004	.055	.066	-		
t	.049	.063	.051	.010	.054	.094**	.063	.063	.006	.105**	.117**	.068	.063	.009	.107**	.057	-.003	.074	.000	-.016	-.012	-.030	.000	-.004	-.048	.020	.038	.006	.355***	-	
u	.076	.083*	.090**	.047	.058	.083*	.087*	.086*	-.020	.035	.043	.037	.038	.061	.021	-.014	.018	-.029	.014	.032	.051	.046	-.007	.008	.007	.015	-.001	-.023	.320***	.440***	-

Note. All significant correlations survived the FDR correction; * $p < .05$; ** $p < .01$; *** $p < .001$. Item numbers correspond to S1 Table and Table 1, main text.
