# Supplementary material for: Affiliation in times of pandemics: Determinants and consequences
Source: PLoS One. 2024 Oct 31;19(10):e0306310. doi: 10.1371/journal.pone.0306310 (PMC11527318; doi:10.1371/journal.pone.0306310)
Supplement: S3 Table — (PDF) [file pone.0306310.s004.pdf]

# S3 Table. Pearson Correlation Matrix for All Study Variables in Sample 2M

| | 1 | 2 | 3 | 4 | 6 | 8 | 9 | 10 | a | b | c | d | c' | d' | e | f | g | h | i | j | k | l | m | n | o | p | q | r | s | t | u |
|---|---|---|---|---|---|---|---|---|---|---|---|---|---|---|---|---|---|---|---|---|---|---|---|---|---|---|---|---|---|---|---|
| 1 | - | | | | | | | | | | | | | | | | | | | | | | | | | | | | | | |
| 2 | .655*** | - | | | | | | | | | | | | | | | | | | | | | | | | | | | | | |
| 3 | .590*** | .686*** | - | | | | | | | | | | | | | | | | | | | | | | | | | | | | |
| 4 | .637*** | .657*** | .693*** | - | | | | | | | | | | | | | | | | | | | | | | | | | | | |
| 6 | .588*** | .599*** | .627*** | .682*** | - | | | | | | | | | | | | | | | | | | | | | | | | | | |
| 8 | .445*** | .471*** | .508*** | .498*** | .542*** | - | | | | | | | | | | | | | | | | | | | | | | | | | |
| 9 | .482*** | .506*** | .564*** | .576*** | .625*** | .785*** | - | | | | | | | | | | | | | | | | | | | | | | | | |
| 10 | .453*** | .508*** | .577*** | .548*** | .564*** | .711*** | .752*** | - | | | | | | | | | | | | | | | | | | | | | | | |
| a | .022 | .067 | .038 | .005 | .010 | .016 | -.017 | .009 | - | | | | | | | | | | | | | | | | | | | | | | |
| b | .164*** | .182*** | .158*** | .127** | .065 | .142** | .117** | .116** | .478*** | - | | | | | | | | | | | | | | | | | | | | | |
| c | .122** | .144** | .100* | .153** | .131** | .155*** | .166*** | .192*** | .284*** | .185*** | - | | | | | | | | | | | | | | | | | | | | |
| d | .093 | .130** | .046 | .163*** | .116** | .129** | .152** | .151** | .221*** | .098* | .720*** | - | | | | | | | | | | | | | | | | | | | |
| c' | .158*** | .174*** | .167*** | .227*** | .273*** | .176*** | .198*** | .188*** | .134** | .076 | .480*** | .395*** | - | | | | | | | | | | | | | | | | | | |
| d' | .078 | .112* | .037 | .153** | .211*** | .128*** | .131*** | .123*** | .109* | .043 | .393*** | .531*** | .672*** | - | | | | | | | | | | | | | | | | | |
| e | .079 | .107* | .081 | .118** | .057 | .026 | .040 | .029 | .240*** | .261*** | .076 | .071 | .112* | .109* | - | | | | | | | | | | | | | | | | |
| f | .082 | .114** | .039 | .106* | .095 | -.026 | .040 | -.008 | .209*** | .161*** | .039 | .086 | .172*** | .130** | .578*** | - | | | | | | | | | | | | | | | |
| g | -.114** | -.111* | -.121** | -.102* | -.128** | -.017 | -.081 | -.077 | .061 | .056 | -.121** | -.075 | -.287*** | -.160*** | -.075 | -.184*** | - | | | | | | | | | | | | | | |
| h | .149** | .135** | .070 | .090 | .145** | .078 | .115** | .109* | .009 | .033 | .082 | .044 | .195*** | .108** | .131** | .275*** | -.449*** | - | | | | | | | | | | | | | |
| i | -.023 | -.046 | -.064 | -.063 | -.083 | -.034 | -.098* | -.117** | .092 | .070 | -.065 | .006 | -.162*** | -.080 | -.100* | -.121** | .483*** | -.407*** | - | | | | | | | | | | | | |
| j | -.042 | -.015 | -.047 | -.009 | -.064 | .035 | -.020 | .002 | .097* | .090 | .000 | .024 | -.134** | -.060 | -.042 | -.166*** | .660*** | -.428*** | .452*** | - | | | | | | | | | | | |
| k | -.068 | -.054 | -.115** | -.085 | -.107* | .016 | -.052 | -.035 | .076 | .088 | .016 | .038 | -.169*** | -.068 | -.099* | -.208*** | .659*** | -.427*** | .481*** | .867*** | - | | | | | | | | | | |
| l | -.074 | -.056 | -.087 | -.083 | -.081 | .037 | -.029 | -.022 | .110* | .073 | -.010 | .022 | -.173*** | -.081 | -.070 | -.158*** | .679*** | -.411*** | .469*** | .866*** | .875*** | - | | | | | | | | | |
| m | -.050 | -.032 | -.074 | -.088 | -.095 | .033 | -.021 | .000 | .119** | .115** | .005 | .007 | -.139** | -.099* | -.069 | -.235*** | .550*** | -.396*** | .403*** | .553*** | .603*** | .600*** | - | | | | | | | | |
| n | -.049 | -.014 | -.082 | -.076 | -.102* | .032 | -.026 | -.019 | .143** | .124** | .015 | .006 | -.143** | -.111* | -.064 | -.196*** | .553*** | -.416*** | .389*** | .561*** | .602*** | .576*** | .922*** | - | | | | | | | |
| o | -.073 | -.026 | -.074 | -.066 | -.100* | .044 | .006 | .019 | .103* | .125** | .020 | .029 | -.115** | -.105* | -.050 | -.189*** | .516*** | -.359*** | .379*** | .543*** | .562*** | .571*** | .872*** | .848*** | - | | | | | | |
| p | -.002 | -.014 | -.058 | -.041 | -.075 | .035 | .004 | .008 | .124** | .079 | -.010 | .023 | -.123** | -.086 | -.007 | -.072 | .376*** | -.234*** | .213*** | .300*** | .334*** | .317*** | .466*** | .472*** | .448*** | - | | | | | |
| q | -.071 | -.033 | -.057 | -.057 | -.091 | .010 | -.028 | .008 | .131** | .113* | -.012 | .023 | -.146** | -.126** | -.063 | -.124** | .497*** | -.317*** | .348*** | .440*** | .465*** | .496*** | .688*** | .711*** | .684*** | .549*** | - | | | | |
| r | -.049 | -.031 | -.091 | -.101* | -.101* | .020 | -.040 | .000 | .145** | .093 | -.017 | -.008 | -.171*** | -.175*** | -.091 | -.145** | .459*** | -.394** | .417*** | .462*** | .481*** | .469*** | .634*** | .649*** | .621*** | .623*** | .692*** | - | | | |
| s | .241*** | .240*** | .196*** | .208*** | .216*** | .238*** | .281*** | .259*** | .006 | .065 | .157*** | .128** | .164*** | .110* | .156*** | .202*** | -.071 | .244*** | -.059 | -.006 | -.042 | -.017 | -.053 | -.068 | -.056 | -.019 | -.046 | -.082 | - | | |
| t | .187*** | .243*** | .190*** | .205*** | .196*** | .157*** | .222*** | .232*** | .082 | .037 | .195*** | .138** | .163*** | .076 | .147** | .209*** | -.121** | .370*** | -.133** | -.045 | -.073 | -.038 | -.114** | -.152** | -.121** | -.059 | -.065 | -.126** | .656*** | - | |
| u | .224*** | .254*** | .201*** | .206*** | .247*** | .197*** | .216*** | .232*** | .051 | .073 | .193*** | .102* | .234*** | .104* | .141** | .239*** | -.184*** | .427*** | -.199*** | -.121** | -.175*** | -.109* | -.181*** | -.228*** | -.178*** | -.078 | -.146** | -.170*** | .661*** | .790*** | - |

*Note.* All significant correlations survived the FDR correction; * p < .05; ** p < .01; *** p < .001. Item numbers correspond to S1 Table and Table 1, main text.
