# Supplementary material for: Affiliation in times of pandemics: Determinants and consequences
Source: PLoS One. 2024 Oct 31;19(10):e0306310. doi: 10.1371/journal.pone.0306310 (PMC11527318; doi:10.1371/journal.pone.0306310)
Supplement: S4 Table — (PDF) [file pone.0306310.s005.pdf]

S4 Table. Pearson Correlation Matrix for All Study Variables in Sample 2W

		1	2	3	4	5	6	7	8	9	10	11	12	13	14	15	16	17	18	19	20	21	22	23	24	25	26	27	28	29	30	31
1	SCO ability item1	-																														
2	SCO ability item 2	.672***	-																													
3	SCO ability item 3	.609***	.732***	-																												
4	SCO ability item 4	.599***	.619***	.691***	-																											
5	SCO ability item 6	.576***	.566***	.647***	.706***	-																										
6	SCO opinion item 8	.462***	.403***	.478***	.481***	.489***	-																									
7	SCO opinion item 9	.454***	.498***	.589***	.563***	.545***	.716***	-																								
8	SCO opinion item 10	.485***	.471***	.549***	.519***	.510***	.684***	.704***	-																							
9	Perceived risk at home	.023	-.020	-.043	-.058	-.046	.025	-.032	.028	-																						
10	Perceived risk for oneself	.091	.013	-.006	-.023	.016	.023	-.033	.034	.567***	-																					
11	Fear during confinement	.173***	.209***	.157***	.127**	.111*	.097	.112*	.112*	.217***	.214***	-																				
12	Anxiety during confinement	.170***	.172***	.150**	.127**	.142**	.095	.120**	.105*	.176***	.197***	.742***	-																			
13	Fear before confinement	.225***	.239***	.221***	.191***	.120**	.083	.162***	.118**	.100*	.050	.457***	.347***	-																		
14	Anxiety before confinement	.141**	.205***	.146**	.131**	.112*	.030	.070	.098*	.139**	.072	.386***	.532***	.583***	-																	
15	Probability of infection	.127**	.121**	.078	.055	.058	.035	.085	.042	.191***	.134**	.141**	.184***	.156***	.166***	-																
16	Probability of symptoms	.150**	.115**	.067	.084	.075	.094	.102*	.117**	.135**	.118**	.094	.120**	.159**	.126**	.592**	-															
17	Declared compliance with the confinement	-.067	-.059	-.087	-.128**	-.137**	.019	-.078	-.067	.148**	.177***	.129**	.037	-.070	-.069	-.050	-.055	-														
18	Number of unauthorized excursions	.107*	.058	.061	.141**	.113**	.050	.046	.044	.009	-.034	.009	-.017	.117**	.058	.052	.217**	-.358***	-													
19	Percent confined time	.020	.039	.005	.010	.029	-.035	-.011	-.033	.119**	.076	.102*	.065	.019	.048	.018	-.086	.441***	-.234***	-												
20	Intentions to comply with authorities (C.)	-.027	-.024	-.030	-.083	-.038	.022	-.002	-.038	.172***	.173***	.125**	.045	-.059	-.048	-.049	-.062	.703***	-.349***	.435***	-											
21	Intentions to comply with the confinement	-.049	-.033	-.030	-.087	-.077	.002	-.033	-.047	.152***	.145**	.069	-.012	-.059	-.082	-.011	-.055	.695***	-.339***	.433***	.848***	-										
22	Willingness to comply with the confinement	-.016	-.032	-.035	-.074	-.035	.024	.002	-.021	.165***	.153***	.112*	.042	-.042	-.035	-.012	-.046	.721***	-.340***	.455***	.911***	.887***	-									
23	Intentions to comply with authorities (PM.)	-.018	-.017	-.073	-.047	-.026	.044	-.014	.013	.091	.106*	.093	.030	-.036	-.034	-.020	-.102*	.519***	-.266***	.319***	.519***	.486***	.524***	-								
24	Intentions to comply with protective measures	-.020	-.018	-.075	-.054	-.040	.033	-.014	-.012	.095	.120**	.096	.040	-.033	-.045	-.005	-.051	.524***	-.225***	.347***	.519***	.506***	.534***	.913***	-							
25	Willingness to comply with protective measures	.004	.021	-.015	-.013	.013	.034	.020	-.001	.054	.087	.074	.045	.000	.011	-.044	-.100*	.442***	-.174***	.246***	.415***	.386***	.467***	.802***	.727***	-						
26	Frequency using protective measures	.002	-.017	-.092	-.049	-.070	.013	-.037	-.013	.086	.101*	.029	.020	-.016	-.018	.005	-.032	.328***	-.115**	.213***	.298***	.291***	.301***	.453***	.440***	.369***	-					
27	Declared compliance with protective measures	-.049	-.057	-.121**	-.091	-.104*	-.068	-.084	-.091	.128**	.116**	.066	.009	-.047	-.103*	-.076	-.128**	.478***	-.194***	.302***	.465***	.410***	.425***	.559***	.529***	.462***	.444***	-				
28	Percent time using protective measures	-.063	-.062	-.095	-.094	-.112*	-.014	-.074	-.069	.124**	.087	.046	-.034	-.075	-.117**	-.056	-.106*	.432***	-.210***	.353***	.378***	.351***	.367***	.573***	.556***	.453***	.613***	.594***	-			
29	Time on social media	.134**	.104*	.092	.107*	.098*	.146**	.148**	.122**	.033	.044	.216***	.152***	.174***	.077	.128**	.135**	.007	.137**	.000	-.014	.011	.023	-.004	.013	.033	.013	.006	.015	-		
30	Time on the phone	.223***	.149**	.153***	.174***	.171***	.206***	.222***	.159***	.045	.015	.214***	.186***	.131**	.021	.150**	.160***	-.024	.250***	-.039	-.060	-.085	-.031	-.024	-.018	-.003	-.044	.018	.001	.585***	-	
31	Time on internet communication	.209***	.162***	.189***	.214***	.187***	.156***	.180***	.150**	.009	.018	.132**	.121**	.116**	.032	.076	.130**	-.108*	.345***	-.062	-.158***	-.155***	-.108*	-.084	-.078	-.045	-.078	-.088	-.091	.479***	.677***	-

Note. All significant correlations survived the FDR correction; * p < .05; ** p < .01; *** p < .001
