# Supplementary material for: Affiliation in times of pandemics: Determinants and consequences
Source: PLoS One. 2024 Oct 31;19(10):e0306310. doi: 10.1371/journal.pone.0306310 (PMC11527318; doi:10.1371/journal.pone.0306310)
Supplement: Attachment — Submitted filename: REBUTTAL.docx [file pone.0306310.s006.docx]

# The Editor

Thank you for submitting your manuscript to PLOS ONE. After careful consideration, we feel that it has merit but does not fully meet PLOS ONE’s publication criteria as it currently stands. Therefore, we invite you to submit a revised version of the manuscript that addresses the points raised during the review process. As you can see from their comments, the reviewers disagree widely, with Reviewer 2 suggesting only minor revisions, and Reviewer 3 recommending rejection. I have read the paper myself, and my thoughts are more in line with Reviewer 1, who thinks that the manuscript has merit, but that there need to be some fairly major revisions to get it to a publishable state. As such, I am inviting the authors to resubmit after making Major Revisions.

We are sorry it took us that long to get back to you. We are extremely grateful for the valuable feedback received from the Editor and the 3 Reviewers. We thoroughly revised the manuscript to accommodate their comments. We now believe our manuscript is much stronger and suitable for publication. Please find below (in blue ink) our response to each of the points raised.

In their revised manuscript, the authors should pay careful attention to all reviewers’ concerns and be clear about how they have addressed those concerns. I will most likely send out the paper again to these reviewers. Please note that I disagree with Reviewer 3’s first concern about the sample collection: this is an important concern about Sample 1, but Sample 2 appears to resolve most of those concerns, which is why I am inviting a revision. Nevertheless, the revised manuscript must still address this concern: acknowledge the potential problems with Sample 1, be explicit about how Sample 2 does or doesn’t resolve those problems, as well as Reviewer 3's other concerns regarding subcultural differences that can’t be investigated and the issue of timing.
The revised manuscript now explicitly acknowledges the main limitation of Sample 1 and how it is addressed with Sample 2 (see lines 713-717). We also address the other concerns of Reviewer 3, specifically citing the limitations of our study, notably concerning subcultural differences and the timing of the study (see the revised Discussion section).

In addition, make sure that it’s clear in all figure captions what the difference is from other figures, for example if it’s the figure for just men or just women. The figure & table captions could also stand to have the description first in plain English for quick reference. Place all figure captions right below the figure with no intervening text. Also, be clear about where in the manuscript it shows that women were more compliant with health guidelines.
All of this has now been done. Table 2 and the accompanying text (lines 573-576) shows that women were more compliant with lockdown restrictions.

1. Please ensure that your manuscript meets PLOS ONE's style requirements, including those for file naming. The PLOS ONE style templates can be found at
<https://journals.plos.org/plosone/s/file?id=wjVg/PLOSOne_formatting_sample_main_body.pdf> and
<https://journals.plos.org/plosone/s/file?id=ba62/PLOSOne_formatting_sample_title_authors_affiliations.pdf>
This has been done.

2. Please provide additional details regarding ethical approval in the body of your manuscript. In the Methods section, please ensure that you have specified the name of the IRB/ethics committee that approved your study.
This has been added (IRB00011540-2020-31).

3. Please provide additional details regarding participant consent. In the ethics statement in the Methods and online submission information, please ensure that you have specified (1) whether consent was informed and (2) what type you obtained (for instance, written or verbal, and if verbal, how it was documented and witnessed). If your study included minors, state whether you obtained consent from parents or guardians. If the need for consent was waived by the ethics committee, please include this information.
This information has been added, line 139.

If you are reporting a retrospective study of medical records or archived samples, please ensure that you have discussed whether all data were fully anonymized before you accessed them and/or whether the IRB or ethics committee waived the requirement for informed consent. If patients provided informed written consent to have data from their medical records used in research, please include this information.
Non-applicable.

Once you have amended this/these statement(s) in the Methods section of the manuscript, please add the same text to the “Ethics Statement” field of the submission form (via “Edit Submission”).
This has been done upon resubmission.

For additional information about PLOS ONE ethical requirements for human subjects research, please refer to <http://journals.plos.org/plosone/s/submission-guidelines#loc-human-subjects-research>.
We now abide by your guidelines.

4. We note that the grant information you provided in the ‘Funding Information’ and ‘Financial Disclosure’ sections do not match. When you resubmit, please ensure that you provide the correct grant numbers for the awards you received for your study in the ‘Funding Information’ section.
Our bad! They now match.

5. We note that you have stated that you will provide repository information for your data at acceptance. Should your manuscript be accepted for publication, we will hold it until you provide the relevant accession numbers or DOIs necessary to access your data. If you wish to make changes to your Data Availability statement, please describe these changes in your cover letter and we will update your Data Availability statement to reflect the information you provide.
The data and analysis scripts can be accessed at: <https://osf.io/p3rtx/?view_only=f7ecdb41adc949e19d921498e4169e76> This has been noted in the manuscript.

6. Please include captions for your Supporting Information files at the end of your manuscript, and update any in-text citations to match accordingly. Please see our Supporting Information guidelines for more information: <http://journals.plos.org/plosone/s/supporting-information>.

This has been done.

# Reviewer #1 (Dr. Jessica D Ayers)

In this manuscript, the authors assess some of the potential factors that may have influenced affiliative needs and compliance with public health guidelines during the COVID-19 pandemic. Specifically, the authors aimed to understand how social comparison orientation, perceived vulnerability to COVID-19, the likelihood of having already had COVID-19, and fear and anxiety during the lockdown influenced affiliative behaviors which, in turn, influenced confinement intentions and behaviors and protective intentions and behaviors in two French samples. The authors report that they found evidence for higher social comparison orientation and higher perceived threat increased the frequency of affiliative behaviors which, in turn, were associated with lower intentions to abide by confinement and protective measures as well as less compliance with these measures. However, this relationship was only found in men in Sample 2. In all, the manuscript presents an interesting case study of the circumstances and cultural changes that occurred during the COVID-19 pandemic. I found the manuscript to be very interesting, well conceptualized, and provides many open opportunities for researchers to examine similar relationships in regions that are still dealing with COVID-19-related restrictions as well as regions that are transitioning to life “post” COVID. However, even with these strengths, I do have some outstanding concerns about this manuscript that I feel the authors would need to address in order for this manuscript to be suitable for publication. I, therefore, recommend a major revision of this manuscript. Below I provide details about my concerns, enumerated for ease of communication during the review process.

Thank you for your clear assessment of our manuscript and for taking the time to offer such constructive comments. We took them all into account in our revisions (see our responses below).

1. The abstract is set up in a logical and straightforward manner, but the presentation of the results in the abstract feels a little off. I think the authors were trying to stay true to the order in which they investigated these questions, but it seems weird as a reader when the authors say that they found an overarching pattern, and then correct themselves and say “this pattern was only found in men.” I would suggest altering this so it is easier for readers to attain the main takeaway from this study.

This was indeed our initial idea but we understand it did not read well. This has been changed according to your suggestion - thanks!

2. There appeared to be a few issues with the English translation of the document. For example, the introduction switches from present tense to past tense a few times between paragraphs, incorrect conjugations of words are used (“analyzes” vs “analyses”), COVID in all lower case letters (when the English usage is in all upper case letters), etc. I do not think that these errors are representative of the authors’ writing, but rather that these are areas that need a little more attention when proofreading.

Thanks for pointing this out. We have asked for proofreading from a native speaker. We now hope our manuscript is free of grammatical errors and inconsistencies.

3. The introduction makes a rather larger deal out of sex differences in some of the COVID research, but more information about these studies is needed to fully understand how these differences are leading to the motivation for the study. For example, the authors write: “They found that women were more likely than men to perceive the Covid-19 pandemic as a very serious health problem, to agree with reinstating public policy measures (social distancing rules), and to report compliance with them” before going on to explain that many factors did not account for the sex differences. But this doesn’t really explain how the importance of sex differences in this research, only that they are not explained. Since there is a decent amount of research on sex differences in response to the pandemic (Ayers et al., 2022; Chan et al., 2021; Giel & Derntl, 2022; Juvonen et al., 2022; Klein et al., 2020; Lippke et al., 2021; Sharma et al., 2020), including with friendships, it seems a little odd that these and other relevant references are not in the manuscript.

We now discuss more clearly the role ‘gender’ differences (note: the use of ‘gender’ is explained in our other responses) could play in response to the pandemic (making use of the very useful references you provided), and explain why this is relevant for the topic of affiliation in stressful situations.

4. I was a little confused as to why the researchers used social comparison as a means to reduce anxiety. I am aware that downward social comparison generally makes people feel better about themselves (Gibbons & Gerrard, 1989) and that this is especially true for those who have lower self-esteem, but this distinction isn’t made in the introduction. It would be helpful here for the authors to further elaborate on these aspects.

We actually measured and used ‘horizontal’ social comparison (but not downward or ‘vertical’ social comparison), as we explain lines 55-58. Horizontal social comparison is linked to social comparison and sense-making under anxiety. This term is now used consistently.

5. There appears to be a citation missing in the first paragraph on page 3. It only states “(2021)” so it is not clear what research the authors are referring to.

This has been corrected – thank you!

6. I do not think that the rationale that these differences are based on gender socialization is well supported in the current version of the manuscript. Much more information is needed here to make this claim, and using only 2 citations without the full rationalization in the introduction, makes me hard-pressed to think that this is actually the case.

One potential implication of this comment is that we may have uncovered a difference between ‘male and female’ which is not the product of gender socialization itself but rather a product of biological differences (and the expression thereof). This is an intriguing possibility, but we are afraid we have no means to know further with our data. We now discuss this possibility (lines lines 695-703), and why we cannot write further on this. We also comment on our use of ‘gender’ (in the Introduction [lines 102-107] and Discussion [lines 695-703]), which may not fully correspond to how the term is widely used. This is justified by the nature of our Sample, which we felt, may not be accustomed to the current conceptualization. Note that other studies (e.g. Galasso et al. 2020, cited in the manuscript) confused ‘gender’ and ‘sex’, probably because the concept of ‘gender’ as self-definition may not be globally accessible.

7. There are some statements in “The Present Research” section that are not well explained. For example, I do not know what “... but also left open the type of influence later considered by Tunçgenç et al., 2021…” and “... the expression of social comparison and related affiliative needs..” actually refers to nor how it sets up this study.

Apologies for these unclarities – this and other parts of the Introduction have been largely revised!

8. Similarly to this, the authors mention that “there is evidence that the strength of this tendency varies between individuals, regardless of gender…” so why make predictions about sex differences then. This seems to act in opposition to the motivation to understand sex differences in these relationships.
We have been using ‘gender’ and ‘sex’ interchangeably here (as we now explain lines 102-107) ­­– see our response to comment 9 also (below).

9. It is not clear why you use “gender differences” and “gender groups” in your writing. Unless you asked participants for self-identified gender and provided many categories or used open-response questions, you are not looking at gender differences. You are looking at sex differences, which fundamentally are not the same thing. Since nowhere in the manuscript is there mention of how broad gender categories were defined from participant responses or how responses in open text fields were dealt with, my assumption is that you did not ask these kinds of questions. Since gender is such a personal thing for most people, I would urge authors to not use this word unless they actually gave participants a comprehensive list of gendered terms to choose from, since the influences and origin of “sex” differences are not the same as the influences and origin of “gender” differences.

We understand Reviewer #1’s concern, but we now explain in several places why we use ‘gender’ and not ‘sex’. We know this is at odd with the concept of ‘gender’ as self-definition which is largely used in the US, and increasingly so in France (where the study was conducted). But using the concept of ‘sex’ could imply we uncovered ‘biological differences’ – which we have no means to evaluate.

10. The explanation for the study is not straightforward (i.e., “the higher social comparison orientation, we reasoned…”). It would benefit the reader to have this relationship presented in a more straightforward fashion.

We revised this section, see from line 81. We hope this is now clearer.

11. There is information in the introduction section about the current research that is far more appropriate for the method section.

The Introduction and Methods section were thoroughly revised – we now hope this reads more clearly.

12. Which social media sites were used to recruit participants? How were they recruited (targeted ads? Snowball sampling?)? How many participants came from each site?

Sample 1 included 1,299 participants recruited through snowball sampling within our social networks. The study was also published on the University’s web site via a direct link to the questionnaire. Thus, anyone having the link could participate. The origin of the responses as to how participants had received the link to the questionnaire was not verified.

This information is now given, see lines 150-155 for sample 1.
For sample 2, see lines 171-173.

13. It is not clear what 14.95 years of education means. In the US, that would be someone who is finishing their sophomore year of college. Is this the same in France? If not, what does this mean? In study 2, 13.08 years is a freshman in college. Is this accurate for France too?

We now explain this, with reference to the number of years of education spent since first enrolment in school in France (at ~7 years old) – see lines 160-161.

14. More explanation in the participant section about study 1 only considering women would be helpful. As it is currently written, that is not clear until describing study 2, and even then is not completely clear.

This is now explained lines 155-159.

15. Can the authors further elaborate on why they collected two samples in a small period of time instead of collecting one sample over the same period of time and working to ensure that the sex ratio in the sample was even?

We first wanted to recruit participants from social media, for funding reasons. When we noticed there was such an imbalance in sex, we sought and obtained appropriate funding and decided to collect a new sample, using a dedicated platform ensuring balance in distribution of participants across the two sexes and representativeness of the French population. We did not want to pretend we did not collect any data yet, and we thought this first Sample (i.e. Sample 1) could be used for analytical comparisons with Sample 2. This is now explained (see lines 142-148).

16. Was any information on participant SES collected? If it was, it would help for this to be reported.

We collected education (number of years) and occupation. Summary statistics are now reported in the ‘Participants’ subsection.

17. The inclusion of Threat-related emotions seems to come out of nowhere in the statistical analyses portion, so it is not clear to me why they were included. It would help if the authors spent more time on this in the introduction, or provided a rationale in the analyses portion, so it does not feel like these were added as afterthoughts. It would also help if the researchers explained why they were considered separately instead of together.

We have now clarified this in the Introduction. As requested, we have now better grounded the necessity to assess threat-related emotions and have justified in why anxiety and fear were assessed jointly and loaded on a single threat-related emotions latent factor (see line 108 onwards).

18. While the table may help the researchers write the paper and make the figures, the table feels extremely unhelpful to a reader. I found myself not being able to fully understand the figures or the tables (even when I looked at them together). I would strongly suggest that the authors completely remove the table and put the appropriate loadings and item names in the figures for ease of reader understanding.

As requested, we have now removed Table 1 and displayed all factor loadings and item names directly in Figures 1 to 6.

19. It would be helpful to add an additional figure that displays only the indirect paths that the researchers assess in their model. As it is written, there is a lot of information that the reader has to hold in their head to understand the explanation of the mediation procedure.

An additional Figure has been added (now referred to as Figure 2). As suggested, Figure 1 now presents the hypothesised direct relations only while Figure 2 presents the hypothesised indirect relations only.

20. The way the measurement invariance is described in the statistical analyses section makes it seem like the authors haphazardly mixed samples to test for invariance and skipped some assessments of invariance to get to the ones that they were interested in. Since I looked at the supplementary materials, I know that this is not the case, but I doubt that many other readers will take this time to investigate it. As such, the writing here needs to be clearer about how invariance was assessed and the steps that the researchers went through.

The statistical analyses sub-section focusing on measurement invariance has been reworked. We now provide a clearer description of the steps we went through, in which sample, and why we did so.

21. More information on the false discovery rate is needed. How often was it applied, and for what analyses? There is no mention of it throughout the rest of the manuscript, so my assumption was that it was not applied or didn’t change the results, but it would help if the researchers to be more transparent about this.

We agree that more transparency is needed. We have now added this information in the analysis section and in the structural comparison section. Furthermore, all Tables focusing on structural comparisons (Tables 3 and 4) indicate that only the significant p values surviving the FDR correction are presented.

22. I would recommend that the researchers include more summary statements after their technical reports of their analyses. I understand the method and analyses they are using, but it was even difficult for me to keep all of the direct and indirect path results organized in my mind. For example, I found this statement to be extremely helpful “If significant, this indirect effect would indicate that more perceived vulnerability fostered more affiliation tendencies due to the fact that individuals’ threat-related emotions increased during confinement.” Statements like these make the results accessible to readers who have not performed these kinds of analyses before, and also make the results clearer for those who have.

We agree that summary statements are quite necessary. We have now added a summary of the results for each model in the main text, in separate paragraphs.

23. Some of the figures ended up within the figure captions. It would help if the figures and captions were all uniform in their presence throughout the manuscript.

Thank you for pointing out these inconsistencies. We have now made sure that the figures’ captions are all uniform across the manuscript.

24. One open question that I have is why did the researchers not consider the model for men in study 1? I understand that the sample is much smaller, but it seems odd to completely disregard their data.

As rightly pointed out by Reviewer 1, the reason for not considering men in sample 1 is that the number of men in that sample (N = 257) was clearly under the minimum required sample size (N = 450) to allow testing the structural model. We have now added this information line 157.

25. I appreciate the bolded arrows in the figures, but really think that the figures would benefit from the inclusion of the loadings and item wording (or really anything else besides “a”, “b”, “c”, etc.).

We agree that the letter labels made the figures hard to decipher. We have now replaced the old labels (“a”, “b”, “c”, etc.) with the actual variable names in all figures. Many thanks for a very useful suggestion.

26. Some of the organization for the results of study 2 are a little confusing. It would help to present all of the analyses for the complete study 2 before assessing 2W and 2M. I found myself confused as to why the results jumped around so much.

Thank you for this suggestion. We have now refined the organization of the results of sample 2 by gathering all analyses common to all participants before examining separate gender models and group comparison analyses. To this end, the measurement invariance results, originally described after the separate sex models, have been moved to the top of the Sample 2 section. We hope that this new organization improves the readability of the results.

27. Can you explain how you achieved measurement invariance through scalar invariance but the paths for men and women are not the same? This was very confusing to me and made me wonder if you had actually achieved measurement invariance.

Perhaps the ambiguity arose from the terminology regarding the different levels of measurement invariance, ‘metric’ and ‘scalar’, sometimes referred to as ‘weak’ and ‘strong’ invariance. First, we have removed the terms ‘weak’ and ‘strong’ and used only the terms ‘metric’ and ‘scalar’. Now, to answer R1’s question, scalar invariance means that the intercept of the manifest (observed) variables, as well as the factor loadings linking the manifest variables to their respective constructs, are equal across groups so that the scales in both groups can be considered psychometrically equivalent. This is different from comparing the magnitude of the associations among latent (unobserved) variables across groups, which is allowed only after the across-groups psychometric equivalence of the scales is established. As Finch (2014) (now cited line 322) wrote: “It is very important to note that measurement invariance does not imply the same level of performance [...] for males and females” (Abstract). Since differences in latent means may exist between males and females even though the constructs are interpreted in the same way, their relationships may also vary, consequently. We examined these variations in path coefficients using a series of Wald tests (Li et al., 1991), as described in Klopp (2019) which compares path coefficients across groups. We have added the following sentence to clarify this point (see line 561 onwards): ‘While the measurement invariance results shown in Table 1 indicate that constructs were indeed interpreted in the same way by both gender groups, group differences in standardized values may have existed at the latent level, including differences in latent means and the magnitude of path coefficients. These differences can be compared after establishing scalar invariance (i.e., gender-related variations at the construct level are therefore not attributable to differences in intercepts and factor loadings across groups; Klopp, 2019; Putnick & Bornstein, 2016).’.

28. Much more detailed information about measurement invariance, the levels of invariance you achieved, and what they mean, is needed in the results section. As it is, the results read like you picked what invariance you wanted to test for and ignored the rest of the categories. (Your supplemental material makes it clear that this is not the case, but even in this document there is not enough information for novice readers to understand what all of the numbers mean).

More information regarding the different levels of measurement invariance and what they mean, in jargon-free language is now present, notably in the Statistical analysis section.

29. More explanation about why you analyzed and report the differences in standardized latent means, regressions, and correlations. It feels like this is a distinction that the authors think is clear, but on the reader’s side, it is not. Explaining the purpose of these analyses and comparisons would be very helpful.

The purpose of examining latent mean differences here is to estimate the extent to which women and men vary on the series of psychological constructs encompassing one’s compliance to pandemic rules (e.g., levels of affiliation tendencies and compliance to safety measures) which values are estimated at a latent level, that is, through minimizing the measurement error of the constructs thanks to the SEM framework. Likewise, the purpose of assessing gender-related variations in path coefficients (i.e., regression and correlation coefficients) is to evaluate the extent to which the associations between the psychological constructs vary between women and men (e.g., whether men’s levels of compliance to safety measures are influenced more strongly by their affiliation tendencies relative to women), again through minimizing the measurement error of the psychological constructs. We have added this information in the statistical analyses section focusing on measurement invariance, which we have now entitled “Comparisons of Structural Relations as a function of Gender”, line 310 onwards.

30. There is some research on sex differences in anxiety and fear brought upon by the pandemic, so it feels odd that this literature is overlooked (Alsharawy et al., 2021; Bigalke et al., 2020; Semenova et al., 2021; Vallières et al., 2022).

Thank you for pointing out this highly relevant literature. We have now included the suggested literature.

31. In the discussion, there is information about other social species avoiding infected conspecifics (lobsters). It feels weird to cite research on lobsters instead of research on other more closely related phylogenetic cousins (for example, <https://www.nationalgeographic.com/animals/article/quarantine-social-distancing-bee-chimpanzees>).

You are correct. The rationale behind using phylogenetically distanced species as an illustration was to show avoidance of infected others could be a very old trait, evolutionarily speaking. We now include a reference to chimpanzees to show that its presence in humans is likely the result of linear (as opposed to convergent) evolution.

32. In the first paragraph on page 30, it is not clear why you say sex differences in risk-taking, coping styles, or willingness to signal as a risk-taker may explain the results in study 2. More explanation is needed.

This is now explicated, see lines 666-671.

33. Since there is research on how the pandemic has influenced friendships/ affiliation, it seems odd that the research is left out of the discussion (Al-Hasan et al., 2020; Ayers et al., 2020, 2021; Cheng et al., 2020; Ellis et al., 2020; Mesurado et al., 2021).

Thank your for pointing this out. We felt those references are interesting, but probably orthogonal to our main research question.

On the whole, I enjoyed this manuscript but do think there are some serious revisions that need to be made before this manuscript is ready for publication. I hope the authors find these comments with the constructiveness with which they are intended.

Those were very constructive comments indeed - and we thank you for providing them. We hoped we addressed them and that you now find our manuscript suitable for publication.

# Reviewer #2

The authors state that all data are fully available without restriction, however a data Availability Statement describing where the data can be found is missing.

We now provide an OSF link with the data and analysis script.

The manuscript still contains some (grammar) mistakes and typos. For instance, on page 19 “confinement (confinement)”, on page 24 “no other indirect effect were significant”, and “))” a few times. Also, please be consistent in using COVID-19 vs. covid-19. Finally, I’d advise the authors to take a close look at the notation of the references, as the style is not always consistent.

We asked for proofreading, checked for consistent wording and appropriate format in the bibliography.

I didn’t have access to the Appendices referred to in the text, as they weren’t included in the file.

We are sorry you experienced difficulties accessing the Supplementary Materials (aka Appendices). We understand that Reviewer #1 could access them. We hope you will find them easily this time.

To be more complete, I’d suggest including the following relevant reference in the section on research on perceived COVID risk for people themselves and close others (e.g., page 2):

De Vries, E. L., & Lee, H. C. (2022). Friend-shield protection from the crowd: How friendship makes people feel invulnerable to COVID-19. Journal of Experimental Psychology: Applied.

This excellent reference is now included – thanks!

# Reviewer #3

1. Sample collection

a. I cannot feel very confident men and women were comparable groups in this data.

We understand your concern. We would be interested in hearing from you about the sort of ‘demographics’ (besides age, education, and occupation, which we measured) you feel could be relevant to collect in the future. For now, what we could do (as we now do: see lines 704-711) is to acknowledge the fact that a demographic element that has not be collected (besides gender) could explain the observed difference better than our partition across the two genders. In the meantime, we are unsure what this element could be, but we mention race, ethnicity and SES.

i. There are potential self-selection concerns for a survey collected online via social media recruitment.

This is very true indeed. This is acknowledged (see lines 712-716). We also discuss how Sample 2 (which, unlike Sample 1, was representative) could help mitigate this methodological issue.

1. Missing demographics and potentially other unobserved characteristics could indicate that the two groups (men and women) were meaningfully different and, thus, incomparable. a. This cannot be addressed with this country’s data; therefore, it does not seem able to be addressed with a revision.

We would be interested in hearing from you about the sort of ‘demographics’ (besides age, education, and occupation, which we measured; besides geography and socioeconomic situations, which we acknowledge –lines 704-711) you feel could be relevant to collect in the future. This could be added to the discussion of limitations of this study, on top of other mentioned variables (see lines 704-711).

2. Given potential self-selection concerns and omitted variable concerns, it seems very unlikely that the sample is representative of France as a whole.

Study 2 presents a representative sample, considering we are limited by ethical considerations and cannot force people who do not want to take part to do so (see lines 715-716).

a. This means a policymaker or other interested party cannot, with a great deal confidence, anticipate behavioral responses to pandemic policy as the population for which the results of this study were found is unidentifiable for meaningful characteristics.

We would be grateful to hear more about unidentifiable meaning characteristics, to include them as potential confounds and write about why and how these could be measured in future work. This could be very valuable to the community – many thanks in advance!

b. The omitted demographics also mean replicability of the sample population would likely prove unfeasible. Any follow-up study intended to replicate, challenge, or demonstrate an evolution in results observed in this study would be unable to do so.

We provide the demographic information we collected (age, education, occupation) for both samples of Studies 1 and 2. A sample with similar distribution of participants across demographics gender, age, education and occupation could be useful to replicate our study. We could never ascertain we did not miss one additional demographics that could explain our results better than the gender dimension, for demographic indices are numerous and cannot all be included.

ii. Could self-reported outcomes be influenced by gender? Might men and women have responded to survey questions differently?

1. If women were more fearful of potential consequences for revealing non-compliant behaviors, then might they have elected to misreport more compliance than men? If so, this could mean that outcomes were not particularly different between men and women.

This is correct and now discussed (see lines 723-732), although this is unlikely given the anonymity of responses, and the fact that anonymity is guaranteed (and explicitly so) in taking part in our study. Besides, there is no way of potentially controlling this issue with any questionnaire data. People can omit information, and there is no way to capture ‘true’ behavior besides using observation (which we could not do since excursions out of the household were limited in France).

2. Depending on unobserved characteristics, could there be subcultural differences in how survey questions were interpreted and answered?

There could be - but answering this question is not easy in France, since we cannot (by law) ask about ethnicity and other ‘race’ identifiers. We now discuss this possibility (lines 704-711) but we are not in position to address this using our data.

2. Timing

a. The data comes from early in the pandemic when the effects of the disease and policy aimed at combatting the disease were largely unknown. Since this time, social understanding, adaptation, and perspectives have evolved substantially.

i. It is unclear how well the findings based on this data would predict behaviors should the threat of COVID-19 escalate to pandemic levels again or a new pandemic arise.

We now briefly speculate on this issue (see lines 733-738).

1. This would suggest the primary contribution of this work would be from the more scientific perspective of whether the relationships between explanatory variables and outcomes were identified correctly.

The primary contribution is to characterize the potential impact of a sociopsychological force (‘affiliation’) on reports of adherence to guidelines during the covid-19 pandemic. We believe this is an honest contribution, which we hope could be useful to a diversity of (academic and non-academic) communities.

Due to such concerns, I am skeptical about the strength of this paper’s contribution. I think the potential policy contribution is limited by timing and data concerns. Further, I believe the social-scientific contribution seems limited by data concerns that cannot be addressed.

We thank Reviewer #3 for their assessment and we hope that our revisions will convince them that our manuscript has, despite clear limitations, certain merits. We believe our manuscript is theoretically sound, honest about its methodology, and statistically correct. Whether it is eventually ‘useful’ is beyond our immediate control now.
